# Continuous but not intermittent noise has a negative impact on mating success in a marine fish with paternal care

**DOI:** 10.1038/s41598-019-41786-x

**Published:** 2019-04-02

**Authors:** Eva-Lotta Blom, Charlotta Kvarnemo, Isabelle Dekhla, Sofie Schöld, Mathias H. Andersson, Ola Svensson, M. Clara. P. Amorim

**Affiliations:** 1 Department of Biological and Environmental Sciences, University of Gothenburg, Box 463, SE-405 30 Gothenburg, Sweden; 2 The Linnaeus Centre for Marine Evolutionary Biology, University of Gothenburg, Box 460, SE-405 30 Gothenburg, Sweden; 3 Department of Marine Sciences, University of Gothenburg, Box 100, SE-405 30 Gothenburg, Sweden; 4 Swedish Meteorological and Hydrological Institute, Folkborgsvägen 17, SE-603 80 Norrköping, Sweden; 5 Swedish Defence Research Agency, SE-164 90 Stockholm, Sweden; 6 School of Natural Sciences, Technology and Environmental Studies, Södertörn University, Huddinge, Sweden; 7 MARE – Marine and Environmental Sciences Centre, ISPA-Instituto Universitário, Rua Jardim do Tabaco, 34, 1149-041 Lisboa, Portugal; 8 Present Address: Departamento de Biologia Animal, Faculdade de Ciências, Universidade de Lisboa, Lisboa, Portugal

## Abstract

Anthropogenic underwater noise is a global pollutant of increasing concern but its impact on reproduction in fish is largely unknown. Hence, a better understanding of its consequences for this important link to fitness is crucial. Working in aquaria, we experimentally tested the impact of broadband noise exposure (added either continuously or intermittently), compared to a control, on the behaviour and reproductive success of the common goby (*Pomatoschistus microps*), a vocal fish with exclusive paternal care. Compared to the intermittent noise and control treatments, the continuous noise treatment increased latency to female nest inspection and spawning and decreased spawning probability. In contrast, many other female and male pre-spawning behaviours, and female ventilation rate (proxies for stress levels) did not differ among treatments. Therefore, it is likely that female spawning decisions were delayed by a reduced ability to assess male acoustic signals, rather than due to stress *per se* and that the silent periods in the intermittent noise treatment provided a respite where the females could assess the males. Taken together, we show that noise (of similar frequency range as anthropogenic boat noise) negatively affects reproductive success, particularly under a continuous noise exposure.

## Introduction

Abiotic and biotic sounds originating from water surface motion, rain, wind and biological communities are natural parts of the marine acoustic environment^1,2^. Organisms living in these habitats use this auditory scene to navigate, find suitable habitats, food, and to avoid predators^3,4^. In addition, many marine animals use acoustic signals to mediate social interactions, such as mate finding, territory defence or predator warning^5–7^. Acoustic cues and signals in water are unique as a sensory modality as aquatic sound propagate with little attenuation over long distances, at all depths, and irrespective of the water current direction^8^.

An additional and growing component in the marine soundscape is continuous broadband noise derived from human activities such as shipping and recreational boats, as well as impulsive sound sources such as pile driving and seismic airguns^9^. Anthropogenic noise therefore creates both constant and temporarily unpredictable fluctuations in the acoustic environment, leaving almost no marine area unaffected^10^. Known impacts on marine organisms range from severe to milder effects^11–13^ depending on noise intensity and temporal patterns of exposure, and on the organisms’ hearing abilities^14,15^. There is growing evidence that marine mammals are affected by noise; noise can mask echolocation and acoustic communication and possibly lead to stranding events^14,16^. In fish, noise can have effects on larval development^17^, foraging success^18^ and predator avoidance^19^. Data on marine invertebrates are still scarce, but point to detrimental effects of noise exposure^20^ including impaired development and increased mortality^21^.

Detrimental effects of noise on reproductive success have been demonstrated in amphibians and birds^22,23^ and recently also in teleost fish^24–26^. Despite an increasing number of studies in fish there is a lack of information regarding the direct impact of noise on fitness, such as mating success or the number of surviving offspring^24^. In the long run, such an impact would affect both individual fitness and population^21^.

Here, we examine how exposure to continuous and intermittent broadband noise affects fitness related traits in the common goby, *Pomatoschistus microps*, a small and very abundant marine fish that plays a relevant role in the coastal ecosystems of the north-eastern Atlantic region, including the Baltic and the Mediterranean Seas^27,28^. Species of the genus *Pomatoschistus* are well-established model systems for bioacoustics and behavioural ecology studies^26,29^. Male common gobies produce a sound as part of their courtship^29^. Thus, acoustic communication between the sexes is likely to be impaired under noisy conditions. Other stereotypical aspects of their reproductive behaviour include male nest-building and nest defence, and paternal care^29,30^. In this study, we quantified key behaviours related to reproduction in the common goby in aquaria under three experimental noise treatments, continuous noise, random intermittent noise and control (no noise added). The measured behaviours were male nest-building, latency to male and female activity (swimming), proportion of time males spent on pre-spawning behaviour (including courtship), latency to female courtship and nest-inspection, female ventilation rate, latency to pairs spawning and probability of spawning. Many of these behaviours can be used as proxies for stress, in particular ventilation rate, but also male nest building and latency to activity by both sexes^31,32^; whereas e.g. delays in female nest inspection, courtship and pair spawning may also result from impeded acoustic communication between the sexes. We predicted that exposure to continuous and intermittent noise would negatively affect both pre-spawning behaviour and mating success, but that continuous noise would be more detrimental than intermittent noise as continuous noise presents a higher cumulative sound exposure level as well as no periods that allow acoustic communication.

## Results

### Male behaviour: nest building and pre-spawning behaviours were not affected by noise treatment in 2015

**Figure 1:**
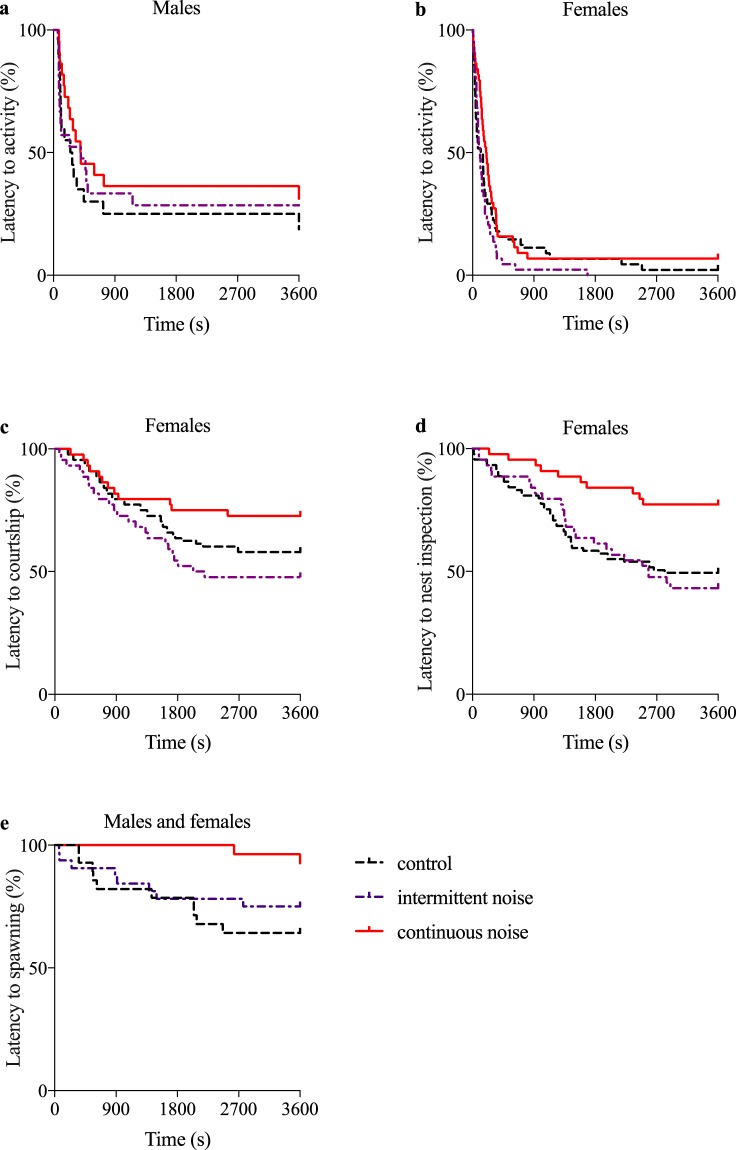
Effect of noise on latency to behaviours in the common goby (*Pomatoschistus microps*). Treatment effects (control, intermittent noise and continuous noise) on (**a**) latency to male activity, (**b**) latency to female activity, (**c**) latency to female courtship, (**d**) latency to female nest inspection and (**e**) latency to spawning. The Kaplan-Meier survival curves show the cumulative percentage of individuals that show a behaviour over time, with 100 indicating that no individual has shown the behaviour. Females in the control and the intermittent noise treatments inspected the male’s nest sooner than females in the noisy treatment (p = 0.003 and p = 0.04). Latency to spawning was significantly shorter in the control treatment compared to the continuous noise treatment (p < 0.01). All other tests were non-significant. control n = 28, intermittent noise n = 32, continuous noise n = 28.

We tested the effect of noise treatment on male nest building effort, based on sand cover after 12 h exposure for single males. No effect of treatment on nest building scores was found (mean ± SD (median); control 1.7 ± 0.9 (1), n = 28; intermittent noise 1.5 ± 0.7 (1), n = 32; continuous noise 1.5 ± 0.7 (1), n = 28; Kruskal-Wallis, χ^2^ = 0.35, p = 0.83). Treatment did not affect latency to male activity (survival analysis, χ^2^ = 1.17, df = 2, p = 0.55; Fig. 1a) or proportion of time spent on active pre-spawning behaviour, including visual courtship (estimated marginal mean (proportion), 95% Wald confidence interval; control 0.07, 0.04–0.13, n = 28; intermittent noise 0.08, 0.04–0.14, n = 32; continuous noise 0.14, 0.07–0.27, n = 28; generalized linear model (GLM), Wald χ^2^ = 1.72, df = 2, p = 0.42).

### Female behaviour: latency to nest inspection increased in continuous noise in 2015

Treatment had no effect on latency to female activity (swimming) after having been released (survival analysis, χ^2^ = 4.47, df = 2, p = 0.11; Fig. 1b) or latency to female courtship (survival analysis, χ^2^ = 2.48, df = 2, p = 0.29; Fig. 1c). However, there was a significant effect of treatment on latency to nest inspection (survival analysis, χ^2^ = 8.74, df = 2, p = 0.013; Fig. 1d). Females in the control and the intermittent noise treatments inspected the male’s nest sooner than females in the continuous noise treatment (survival analysis, control vs continuous noise χ^2^ = 8.57, df = 1, p = 0.003; intermittent vs continuous noise χ^2^ = 4.18, df = 1, p = 0.04; control vs intermittent noise χ^2^ = 0.15, df = 1, p = 0.28). In addition, treatment had no effect on female ventilation rate (ANOVA mean ± SD gill movements per minute; control 64.0 ± 9.2, n = 27; intermittent noise 61.0 ± 11.1, n = 32; continuous noise 60.2 ± 8.9, n = 27; F_2,83_ = 1.00, p = 0.37),

### Latency to spawning increased in continuous noise in 2015

 When analysing if spawning occurred and was documented on video (i.e. the spawning occurred within the first 60 min), treatment had an effect on latency to spawning (survival analysis, χ^2^ = 6.42, df = 1, p = 0.04; Fig. 1e), such that pairs in the control treatment spawned significantly sooner and more often than pairs exposed to continuous noise (survival analysis, χ^2^ = 6.99, df = 1, p < 0.01). However, there was no significant difference in time to spawning between intermittent and continuous noise (χ^2^ = 3.43, df = 1, p = 0.06) or between the control and intermittent noise treatments (χ^2^ = 0.64, df = 1, p = 0.42).

### Mating success decreased in continuous noise in 2015 and 2018

**Figure 2:**
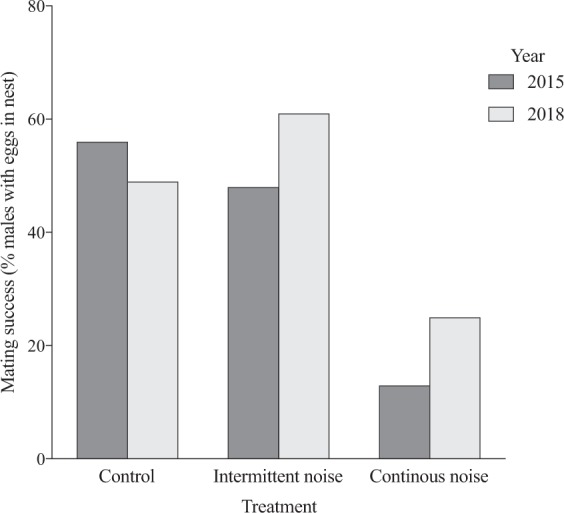
Effect of noise on mating success in the common goby (*Pomatoschistus microps*). Treatment effects (control, intermittent noise and continuous noise) on mating success in year 2015 and 2018 measured as the percent of males receiving eggs. A significantly higher number of males received eggs in both the control treatment (2015: 19 out of 34 males; 2018: 33 out of 67 males, p < 0.001) and the intermittent noise treatment (2015: 16 out of 33 males; 2018: 17 out of 28 males, p < 0.001) compared to the continuous noise treatment (2015: 4 out of 31 males; 2018: 16 out of 65 males). However, there was no significant difference between the control and the intermittent noise treatments (p = 0.64) or between years (p = 0.43).

When analysing mating success, measured in both 2015 and 2018, we found a significant effect of treatment on the proportion of males receiving eggs (estimated marginal mean, 95% Wald confidence interval; control 0.51, 0.41–0.60, n = 101; intermittent noise 0.54, 0.42–0.66, n = 61; continuous noise 0.20, 0.13–0.29, n = 96; GLM, Wald χ^2^ = 31.9 df = 2, p < 0.001), and no difference between years (estimated marginal mean, 95% Wald confidence interval; 2015: 0.38, 0.28–0.48, n = 98; 2018: 0.43, 0.35–0.52, n = 160; GLM, Wald χ^2^ = 0,62 df = 1, p = 0.43). Post hoc tests (LSD) showed a significantly higher number of males receiving eggs in both the control treatment (p < 0.001) and the intermittent noise treatment (p < 0.001) compared to the continuous noise treatment. However, there was no significant difference between the control and the intermittent noise treatments (p = 0.64) (Fig. 2).

## Discussion

In this study, we found detrimental effects of broadband noise on mating success in male and female common gobies. Specifically, testing different noise regimes, we found that continuous noise increased latency to female nest inspection, latency to pair spawning, and decreased spawning probability, compared to the other treatments. Other aspects of male behaviour (nest-building, latency to male activity and proportion of time spent on male pre-spawning behaviour) and female behaviour (latency to female activity, latency to female courtship and ventilation rate) remained apparently unaffected. Detrimental effects of noise on parental behaviour^24^, egg cortisol levels^25^, male courtship and spawning success^26^ have recently been demonstrated in teleost fish. By studying both females and males, our study adds further evidence of a direct impact of aquatic noise on mating success in teleost fish and highlights a difference in severity between intermittent and continuous noise. Furthermore, our results suggest that female spawning decisions were delayed due to a reduced ability to assess male acoustic signals. That said, courtship and spawning are pair activities and it is therefore difficult to ascertain which sex (if any) was more affected by the noise. However, at least based on our chosen endpoints, it appears that the choosing part (females) in the communication dyad, was more affected by noise treatment than the chosen part (males).

Latency to spawn increased with noise exposure and fewer pairs spawned in the continuous noise treatment compared to the control and the intermittent noise treatment. Since males built nests and courted similarly across treatments, this suggests that females were more affected by noise than males were. In fact, females in the noisy treatment had a longer latency to nest inspection, which is an important precursor to spawning. However, all other potential proxies of stress in females (e.g. ventilation rate and latency to activity) did not differ between the treatments. Therefore, we suggest that the noise hampered the pairs’ acoustic communication, which is an important part of courtship in this species^29^. If females are unable to assess male condition or other qualities revealed by courtship sounds^33^, due to continuous noise, they would likely either not spawn, or delay spawning while gathering more information on male quality through other cues. Congruently, in another species of the same genus, the sand goby (*Pomatoschistus minutus*), females appear to only start egg laying after the male produces courtship sounds^34^. In addition, continuous noise decreases acoustic signalling in breeding males in the painted goby (*Pomatoschistus pictus*)^26^. Therefore, we interpreted results as female spawning decisions being delayed by a reduced ability to assess male acoustic signals^26,35^, rather than due to stress *per se*.

An increased time to spawning carries costs to both sexes. Because both males and females spawn repeatedly during their short single season^36^ an increased interspawning interval decreases both their reproductive rate and lifetime reproductive success. Gobies are also food for other fish, and arguably, a prolonged period of mate search and courtship is likely to expose the gobies to predators for a longer time, and hence increase the risk of predation^37^. Curbed communication may also carry costs if females spawn with males they would not have chosen otherwise. This has been suggested to be the case in the sand goby during turbid conditions when visual communication is hampered^38^. In *Pomatoschistus*, courtship sound has been shown to correlate with male condition^33^, which in turn correlates negatively with filial cannibalism i.e. positively with hatching success^30^. Therefore, sub-optimal female choice could decrease not only indirect (genetic) benefits but also direct benefits in the form of paternal care and reproductive success.

In this study, we investigated the effects of noise pattern, by comparing continuous and intermittent noise. We found that continuous noise was detrimental for latency to female nest inspection, latency to spawning and for mating success, whereas this was not the case for the irregular intermittent noise. Masking of the acoustic communication may at least partially explain why continuous noise had a higher impact on the common goby than intermittent noise. As fish in the intermittent treatment had silence 50% of the time, it is likely that females had a chance to assess the male through acoustic cues during this time. In contrast to our results, Nichols and colleagues^39^ found that intermittent noise (speaker playback of boat noise of similar frequency range as in the present study) caused a greater stress response (cortisol concentration) than continuous noise on juveniles of the giant kelpfish (*Heterostichus rostratus*), and suggested that variability in the acoustic environment was a likely for the effect. In contrast, we did not find evidence that common gobies were stressed or otherwise sensitive to the noise exposure treatment, except for potential masking of courtship sounds. Besides studying different species, age and context^32^ the difference in results between our studies might be due to methodological differences. For example, the acclimation (20 h) in Nichols *et al*.^39^ differed from the present study (36 h for males, 1 h for females), and they had considerably less noise exposure (noise 5–12 s and off-time 1-120 s ) than in our study (noise 20–600 s, with a total off-time of 50%). Hence, the long total exposure in our study may have caused habituation^32,40^, and the relatively long periods of up to 10 minutes of noise may also have lessened the effects of unpredictability of the intermittent noise exposure^41^.

Despite the fact that the noise used in our study was broadband and of similar frequency range as boat noise (with most energy below 1 kHz)^19,42^, it is important to note that noise experiments carried out in aquaria cannot reliably mimic exposure to real boat noise in nature. The acoustic field is more complex and particle motion, a component in sound waves that fish and invertebrates are sensitive to^40^, occurs in a more complex pattern in aquaria than in the open sea^43^. Nonetheless, as studies carried out in nature present challenges regarding both control and manipulation of experimental conditions, complementary approaches including laboratory based studies have the potential to increase scientific progress^44^. In addition, a recent comparison between indoor and outdoor noise experiments on the European sea bass (*Dicentrarchus labrax*)^45^ suggests that similarities can be found between nature and laboratory studies. Although our findings should not be directly extrapolated to fitness consequences in nature, they represent evidence of the impact of noise exposure on fish mating success and highlight the need to examine the effects of man-made noise on fish behaviour and reproduction.

In conclusion, our study shows that continuous noise can have an even broader impact on teleost fishes than previously appreciated^17,19,45,46^, affecting the reproductive success of adult fish, and that silent periods in the intermittent noise treatment may provide a respite. Still, more work is needed in the future to examine how different sources of anthropogenic noise may affect reproductive success in different species of fish in nature.

## Materials and Methods

### Study species

The common goby, *Pomatoschistus microps* (Krøyer), is a small (3–6 cm)^47^ marine fish distributed in lagoons, coastal areas and estuaries. Over the course of their single breeding season these short-lived fish (1–2 years) can reproduce repeatedly with different mates^36^. The species is characterized by male-male competition, female choice and a resource-based mating system where males use mussel shells or similar substrates as nest material^48^. Males attract females by visual courtship and lead the female to the nest^29^. If the female enters the nest, the male produces sound, suggesting it functions as vocal courtship^29^. The female leaves the nest after spawning and the male provides exclusive paternal care until hatching^36^. The courtship sound contains a series of low frequency pulses at ~180 Hz^49^ which potentially can be overlapped and masked by many anthropogenic sound sources^4^. It is not fully understood why male gobies produce sound during courtship, but it is likely it carries information that allows the female to assess male condition or other qualities^29,33,50^.

### Experimental design

The experiment was conducted at Sven Lovén Centre for Marine Infrastructure Kristineberg on the west coast of Sweden (58°15′N, 11°27′E) between May and July 2015 (all aspects of the study) and May to August 2018 (mating success only). All fish were caught by hand trawling at a depth between 0.2 and 0.5 m in bays nearby the station. The fish were housed in 50 L storage tanks and separated by sex, for ≥7 days before the experiment started. We conducted the whole experiment in an outdoor greenhouse which guaranteed natural light conditions. All aquaria had a continuous flow of oxygen saturated (fully or slightly over saturated) natural seawater (salinity 22–31 ppt), and water temperature was measured daily (2015: 11–12 °C, 2018: 11–16 °C). The fish were fed every second day with commercial fish food granules (Nutra HP, Skretting) and frozen *Artemia* sp.

We used 30 experimental aquaria (20 L). To insulate the aquaria from ground borne vibrations, each aquarium was placed on top of a 20 cm high rubber cylinder (2015) or wooden planks (2018) on a drainage bench. In both years, the treatments were run simultaneously, but on different benches to avoid interference. Aquaria were separated by opaque screens, to avoid visual interaction between fish in adjoining replicates.

Each aquarium contained sifted sand and was equipped with half a clay flower pot (Ø 65 mm) as an artificial nest site. The pots were fitted with a plastic sheet lining the ceiling for females to lay eggs on. We measured total body length of the fish to the nearest mm (2015: males: n = 88, L _T_ (mean ± SD): 41 ± 4.0 mm; females n = 176, L _T_: 40 ± 3.8 mm, 2018: males: n = 159, L _T_: 40 ± 3.8 mm; females n = 318, L _T_: 41 ± 4.0 mm) before trials.

**Figure 3:**
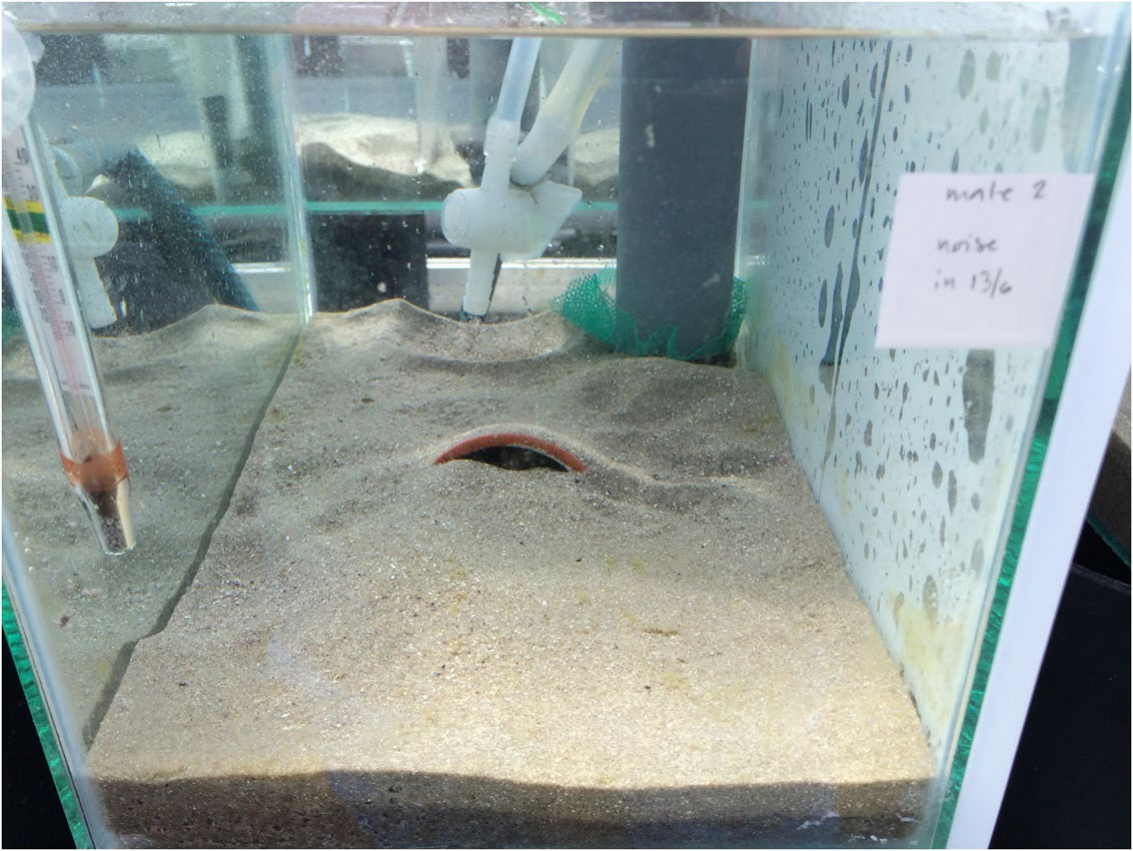
Experimental setup used to test noise effects on common goby (*Pomatoschistus microps*) behaviour and reproductive success. The nest is in the middle of the aquaria with a male inside. A polypropylene tube, whit a lid in the bottom held in place by a net, in the right rear corner of the aquarium was filled with soft airgun balls. The airgun balls were bubbled with air in the noise treatments, either with a random intermittent or with a continuous temporal pattern. In the control treatment, the aeration of the airgun balls was always turned off.

To create artificial noise, we placed an enclosed polypropylene tube (Ø 56 mm) filled with 0.1 l of soft airgun balls, vertically in the right rear corner of each aquarium (Fig. 3) and tumbled the soft airgun balls by bubbling compressed air through an airstone at the bottom of the tube. Apart from being inexpensive, advantages were avoiding electromagnetic fields from speakers which would have required an additional control^21^ and that the noise did not transmit to control tanks. Because of the continued flow of oxygen saturated water into the experimental tanks and that the airstone was inside the tube and not in direct contact with the water in the aquarium, the treatments were not expected to affect oxygen levels. This was confirmed in 2018 when we measured oxygen saturation it in a subset of the replicates in the control and continuous noise treatments. The oxygen levels were typical of the set-up (mean ± SD, control 103 ± 6%, continuous 106 ± 4%) and did not differ between the treatments (t-test, t = 1.30, df = 29, p = 0.20).

In the continuous noise and the control treatment the artificial noise was always on or off, respectively. In the intermittent noise treatment, the air pump was controlled by a timer that was programmed to create random on and off periods ranging between 20–600 s with a total on-time of 50%. All treatments were exposed to low levels of airborne ambient noise, mainly caused by seagulls, wind, and human activities outside the greenhouse. When the fish were in the experimental aquaria they were exposed to the assigned treatment the entire time, but undisturbed by humans between measurements.

Males were randomly assigned to the three experimental treatments (control, intermittent and continuous noise), in the evening. The following morning, we took a picture of each nest for later estimates of nest building. After a total exposure time of 36 h, each male was presented a transparent plastic cup with two ripe females. The females were left in the cup for 1 h to acclimatise before they were released in the aquarium and allowed to interact freely with the male for 12 h. Behavioural interactions were recorded the first 60 min after release with a camcorder (Canon Legria HF M56, Ōta, Tokyo, Japan) placed in front of the aquarium at a 90 cm distance. After 12 h the females were removed, and the nest was examined for eggs. New replicates were started in aquaria as they became available.

All fish used in the experiment were released into their natural environment after the trials. This study complies with the Swedish law and Animal Behaviour guidelines for the treatment of animals in behavioural research and teaching. All experimental protocols were approved by the Gothenburg Ethical Committee on Animal Research (Permit Numbers Dnr 2013-86, Dnr 5.8.18-03920/2018).

### Acoustic measurement

**Figure 4:**
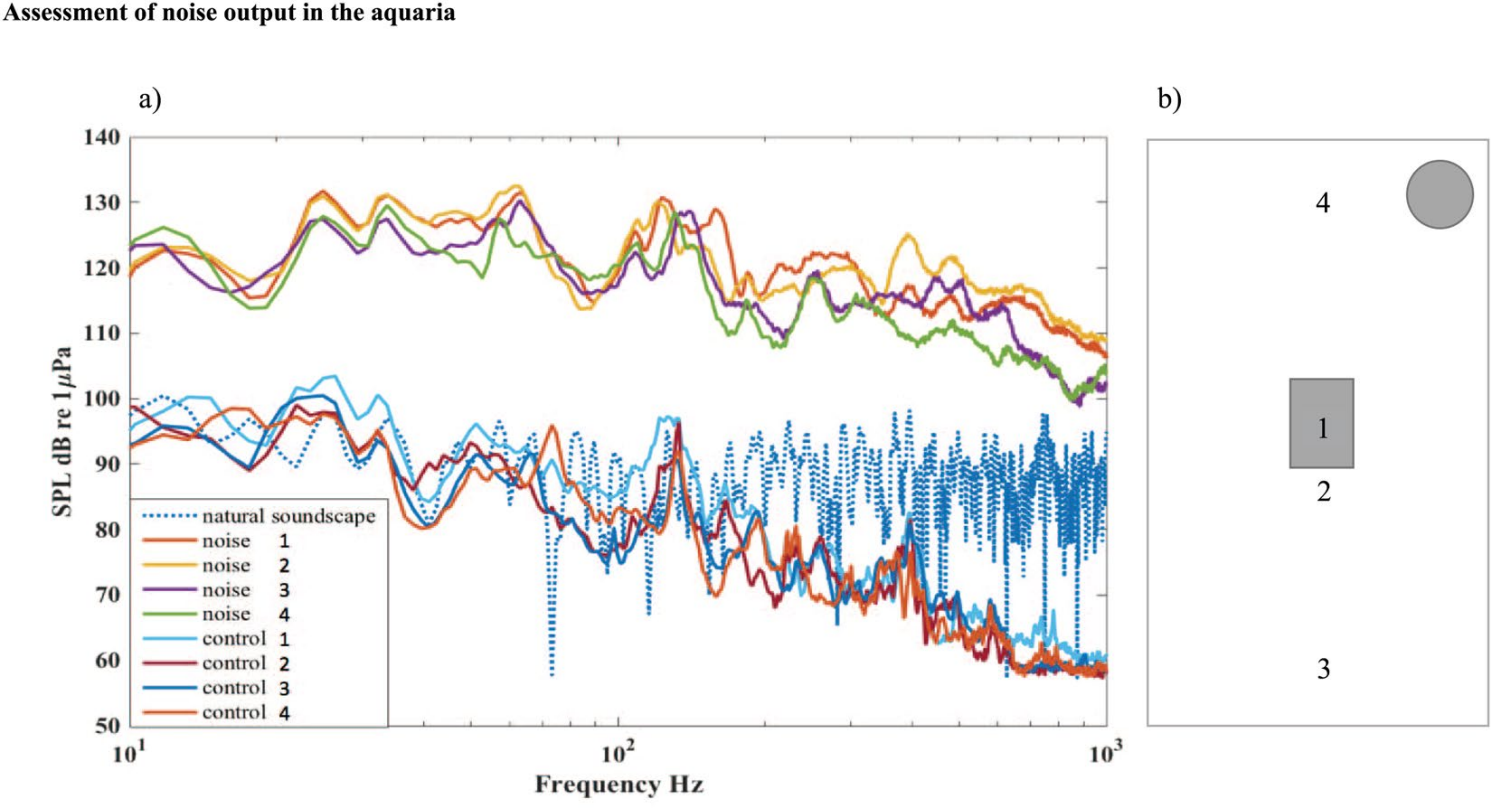
Assessment of noise output in the aquaria. (**a**) Power spectra for noise and control treatments shown for 0–1 kHz. Sound pressure level was on average 34 dB higher for noise than for control in this frequency range (36 dB for 0–12 kHz). For comparison, we have also included the sound spectrum recorded in the bay where fish were collected (natural soundscape). (**b**) Map of sound measurements 2015 within the aquaria. Noise levels were measured with a hydrophone, placed at four different locations in the experimental aquaria: (1) in the nest, (2) 10 cm in front of the nest, (3) 20 cm in front of the nest and (4) is 10 cm behind the nest, near the sound source (marked as a round grey circle). The four lines shown in (**a**) represent each of these four positions. All measurments were done without fish in the aquarium.

Within the species hearing range (<1 kHz)^51^ the artificial noise generated by this system had elevated energy on average 34 dB higher than the control (root-mean-square sound pressure levels, SPLs) (Fig. 4). The noise was of similar broadband character as many anthropogenic noise sources (e.g. boat noise^42,52^). Figure 4 depicts the power spectrum measured under noise and control conditions at four locations inside the experimental aquaria. Sound was registered using a calibrated hydrophone (HTI-96-MIN with pre-amplifier, High Tech Inc., Gulfport MS; sensitivity −165 dB re 1 V/μPa, frequency range 0.02–30 kHz) connected to a digital audio recorder (Song Meter SM2+, Wildlife Acoustics Inc., Maynard, US, sampling frequency 24 kHz). Note that the frequencies of interest in the noise treatment should be unaffected by tank properties as they fall well below its resonant frequency (4.9 kHz)^53^. For comparison, we recorded the natural soundscape close to the bottom (at 0.5 m depth) in the bay where fish were collected using the above equipment with the same recording setting (gain level). Noise in the control treatment had similar or less energy than the natural soundscape (Fig. 4).

### Data analysis

Nest building effort by the male can be scored based on how well the nest site (flower pot, mussel shell, or similar) is covered with sand^30,54,55^. In this study, nest building was judged visually from photos taken after the first night when the male was alone, and it was scored as follows: 1 = the male had piled no sand on the pot, 2 = the male had piled sand on the pot but not covered it and 3 = the pot was completely covered with sand.

We measured the time of active pre-spawning behaviour by the males and time to male and female activity from the videos for the first 20 min using an event recorder (JWatcher + video 1.0; http://www.jwatcher.ucla.edu) and scored the duration of male and female behaviour. The observers were blind to the treatment. A pilot analysis showed that the proportion of time males and females were active during this subset of time did not differ from the whole hour. We defined ‘male active pre-spawning behaviour’ as either male courting the females (with fin displays inside or outside the nest, or with lead swim towards the nest) and as nest building. We defined ‘female active pre-spawning behaviour’ as females actively swimming to males and, presenting her belly (see Blom *et al*.)^29^ for a description of male and female behaviours). In addition, we measured latency to female courtship and nest inspection (i.e. the time it took for females to start courting the male and time to nest inspection) and female ventilation rate (number of gill movements per unit time). The number of female gill movements was counted for 30 seconds. We also measured the time to spawning for the fish that spawned within the 60-min video recording, which in this context is the moment when one of the females started to lay her eggs in the male’s nest. The pairs that spawned after 60 min, and therefore were not caught on video, were included in the analysis as non-spawners. Ventilation rate was used as a proxy for stress, because stress typically results in an increased demand of oxygen in response to increased metabolism, and to meet this increased demand, the heart has to supply more blood per unit time, which then has to be matched by an increased ventilation volume^31,32^. A drawback with ventilation rate is that it might underestimate total oxygen supply, since the latter is also affected by volume per gill movement, but an important benefit is that it can be measured from video recordings. Other behaviours, such as latency to activity by both sexes and nest building behaviour by males, may also reflect stress^32^ whereas delayed nest inspection, courtship or spawning may result either from stress or from impeded acoustic communication between the sexes.

### Sample sizes and statistical analysis

In this study, we had 98 replicates collected in 2015, and 159 replicates collected in 2018, to measure mating success (based on presence or absence of eggs in the nests).

Number of replicates per treatment and year was: control treatment - 2015: 34 males; 2018: 67 males, intermittent noise treatment - 2015: 33 males; 2018: 28 males and continuous noise treatment - 2015: 31 males; 2018: 65 males. We used a subset of 88 replicates from 2015 for the behavioural analyses extracted from the 60-minute video recordings (control; males n = 28 females n = 55, intermittent noise; males n = 32 females n = 64, continuous noise; males n = 29 females n = 54), although in a few cases for females from 2015, the n-values were slightly lower than that, as explained below.

In the statistical analyses of female behaviour, for each replicate we used the average for the two females. However, when analysing female ventilation rate (gill movements per unit time), we were not able to analyse all females e.g. because some were hiding in the sand. In these cases, the value of one female was used. However, in two replicates no females were possible to analyse. Female gill movements were normally distributed and analysed with an ANOVA. Male nest building effort (based on scored values of sand cover) was analysed with Kruskal-Wallis ANOVA. The duration of male active pre-spawning behaviour (including courtship) was analysed using generalized linear model (GLM) with a binomial distribution (robust estimator, link function logit). Male latency to activity as well as female latency to activity, courtship and nest inspection and latency to spawning were analysed using survival analyses with log-rank (Mantel-Cox) tests. We compared time to event (e.g. spawning) between treatments, considering the replicates without the measured behaviour as ‘no event’. Mating success (number of males that received eggs or not) was analysed with a GLM with binomial distribution (model-based estimator, link function logit) and treatment and year as factors. A non-significant interaction between treatment and year (p = 0.29) was removed from the model. GLMs are reported with estimated marginal means and 95% confidence intervals. Significant GLM tests were followed by pair-wise LSD post hoc tests and significant survival analyses by pairwise comparisons.

All statistical analyses were conducted in SPSS (IBM SPSS Statistics for Windows, Version 22.0. Armonk, NY: IBM Corp.), except for the survival analyses, which were performed using GraphPad Prism (version 6.0 h for Mac OS X, GraphPad Software, La Jolla California USA, www.graphpad.com). All model assumptions were met.
